# Ophthalmic Dispensary in Toronto

**Published:** 1868-03

**Authors:** 


*Ophthalmic Dispensary in Toronto.*—This institution was opened last May, and during the first six months there was an average daily attendance of fifteen patients. It is simply a dispensary, no beds or hospital accommodations are yet provided. It is organized under a board of directors, chosen by the benevolent who have contributed to its support. The medical staff under its present organization is as follows:—Surgeon, A. M. Rosebrugh, M. D.; Assistant Snrgeon, R. A. Reeve, M. D.; Consulting Surgeon, W. H. Cumming, M. D. The success of its first year's operation shows the beneficence of the design and the faithfulness and fidelity of those to whom its interests have been entrusted.

